# Prevalence and Predictors of Vitamin D Deficiency among African Immigrants Living in Australia

**DOI:** 10.3390/ijerph16162855

**Published:** 2019-08-10

**Authors:** Kahlea Horton-French, Eleanor Dunlop, Robyn M. Lucas, Gavin Pereira, Lucinda J. Black

**Affiliations:** 1 School of Public Health, Curtin University, Kent Street, Bentley, WA 6102, Australia; 2 National Centre for Epidemiology and Population Health, Research School of Population Health, Australian National University, Acton, ACT 2600, Australia; 3 Centre for Opthalmology and Visual Science, University of Western Australia, 35 Stirling Highway, Crawley, WA 6009, Australia

**Keywords:** vitamin D deficiency, 25-hydroxyvitamin D, African, immigrant, Australian Health Survey

## Abstract

Vitamin D deficiency (serum 25-hydroxyvitamin D (25(OH)D) concentrations <50 nmol/L) is a public health issue in Australia and internationally. Those with darker skin require a greater dose of ultraviolet B radiation from sunlight than those with paler skin to synthesise adequate amounts of vitamin D. Using data from the 2011–2013 Australian Health Survey, we investigated the prevalence and predictors of vitamin D deficiency in African immigrants aged ≥18 years living in Australia (*n* = 236). Serum 25(OH)D was measured using a liquid chromatography–tandem mass spectrometry method that is certified to international reference measurement procedures. Poisson regression was used to investigate independent predictors of vitamin D deficiency. A total of 36% of adults were vitamin D deficient (35% of men, 37% of women). The prevalence ratio (PR) of vitamin D deficiency decreased by 2% per year of age (PR 0.98; 95% CI (0.97, 0.99); *p* = 0.004) and was 1.6 times higher in those with low/sedentary, compared to moderate/high, physical activity levels (PR 1.64; 95% CI (1.12, 2.39); *p* = 0.011). The greatest risk was for those assessed during winter/spring compared with summer/autumn (PR 1.89; 95% CI (1.33, 2.64); *p* < 0.001). Culturally appropriate messaging on safe sun exposure and dietary vitamin D is warranted in order to promote vitamin D sufficiency in African immigrants living in Australia.

## 1. Introduction

There is a high prevalence of vitamin D deficiency worldwide [1], and, despite living in a relatively sunny country, nearly one in four Australian adults are vitamin D deficient (defined as serum 25-hydroxyvitamin D (25(OH)D) concentrations < 50 nmol/L) [2]. The risk is higher for those born outside of Australia and other main English-speaking countries [3]. Previous studies have shown a high prevalence of vitamin D deficiency among African people living in Australia (27% to 99%, depending on season and latitude) [4,5,6,7,8,9,10], and this is an increasing proportion of the total Australian population [11,12]. Furthermore, immigrant children, particularly those with dark skin, have a higher incidence of vitamin D deficiency rickets than the general Australian paediatric population [13]. Daily hours of sunshine and the intensity of ultraviolet B (UVB) radiation during non-summer months in parts of Australia (e.g., southern regions) are lower than some African immigrants’ countries of origin [4], and those with darker skin require a larger dose of UVB to synthesise adequate amounts of vitamin D than those with paler skin [14]. However, current sun-safety guidelines recommend that all Australians, including those with naturally dark skin, protect their skin from sun exposure when the UV index is ≥3 [15,16]. There are currently no sun-safety guidelines specific to Africans.

Previous studies of vitamin D status among African people living in Australia have been relatively small, conducted in single regions/cities, and none of them used an internationally certified assay to measure serum 25(OH)D concentrations. The 2011–2013 Australian Health Survey (AHS) was the first nationally representative survey to quantify serum 25(OH)D concentrations in the Australian population [17]. The AHS used an assay certified to the reference measurement procedures developed by the National Institute of Standards and Technology, Ghent University and the US Centers for Disease Control and Prevention (hereafter referred to as RMPs) [18,19]. Using data from the AHS, we aimed to determine the prevalence and predictors of vitamin D deficiency in a sample of adult African immigrants living in Australia.

## 2. Materials and Methods

The 2011–2013 AHS comprised the National Health Survey (NHS) (*n* = 20,425), the National Nutrition and Physical Activity Survey (NNPAS) (*n* = 12,153) and the National Health Measures Survey (NHMS) (*n* = 10,401). Further details on the AHS can be found elsewhere [20]. In brief, individuals who participated in the AHS provided core information, including general household and demographic data, self-reported health, smoking status, height, weight and information on selected health outcomes. Participants of the AHS were randomly selected to complete either the NHS or the NNPAS. The NHS collected health and medical information, and the NNPAS collected data on physical activity and diet. Data were collected by face-to-face interview with a trained Australian Bureau of Statistics (ABS) interviewer. All participants aged ≥18 years were invited to take part in the NHMS, which involved collection of blood samples for measurement of biomarkers, including serum 25(OH)D concentrations. Blood collection was conducted year-round, with each person sampled on one occasion only. The AHS was conducted in accordance with the Declaration of Helsinki. Interview data were collected under the Census and Statistics Act 1905. For the NHMS component, the Australian Government Department of Health and Ageing’s Department Ethics Committee provided ethics approval in February 2011, and written informed consent was obtained from participants.

This study includes data on individuals aged ≥18 years who were born in Africa (including sub-Saharan Africa, North Africa and the Middle East), participated in the NHMS and had complete data for all potential predictors investigated. A total of 265 participants who were born in Africa had data for serum 25(OH)D concentrations. Of these, 13 participants had missing data for potential predictors (body mass index (BMI), *n* = 8; education, *n* = 4; physical activity, *n* = 1), leaving 236 participants for the current analysis.

The AHS participated in the Vitamin D Standardisation Program (VDSP), which was established to enable international standardisation of serum 25(OH)D concentration measurements [18]. Blood samples were stored at −80 °C prior to analysis by a liquid chromatography–tandem mass spectrometry (LC–MS/MS) method that is certified to the RMPs (Douglass Hanly Moir Pathology, Sydney, Australia). Vitamin D deficiency, insufficiency and sufficiency were defined as serum 25(OH)D concentration <50, 50 to <75 and ≥75 nmol/L, respectively [21].

Region of birth was defined as “sub-Saharan Africa” or “North Africa and Middle East”. Socioeconomic status was scored according to the 2011 Index of Relative Socioeconomic Disadvantage (IRSD). This index is used to summarise information relating to the social and economic resources of people and households within an area using a scoring system ranging from low to high. A low score on the IRSD indicates relatively greater overall disadvantage. IRSD was categorised as low (lower five deciles) or high (upper five deciles). Education was described as “university” or “less than university”. Smoking status was defined as “current smoker” or “ex/non-smoker”. Body mass index (BMI) was calculated from measured weight (kg) and height (m) (weight/height^2^). BMI was classified as underweight (<18.5 kg/m^2^), healthy weight (18.5 to <25 kg/m^2^), overweight (25.0 to <30 kg/m^2^) or obese (≥30 kg/m^2^) [22]. The season of blood collection was defined as “summer” (December to February), “autumn” (March to May), “winter” (June to August) or “spring” (September to November) and further categorised as summer/autumn and winter/spring.

The ABS defined physical activity level according to the duration and intensity of activities undertaken for fitness, sport or recreation and transportation in the past seven days [20]. Activities were categorised and assigned an intensity factor as follows: walking for fitness or transport = 3.5, moderate exercise/physical activity = 5.0 and vigorous exercise/physical activity = 7.5. The intensity factor was applied to the duration (minutes) of the activity to derive a score, which was categorised as “low/sedentary” (<800), “moderate” (800–1600, or >1600 with <1 h of vigorous physical activity) or “high” (>1600 with ≥1 h of vigorous physical activity). We further categorised physical activity as “low/sedentary” and “moderate/high”.

Characteristics of participants were described using number and proportion for categorical variables in the total population (*n* = 236) and in those with vitamin D deficiency (*n* = 85) and vitamin D sufficiency (*n* = 151). All continuous variables had a normal distribution; hence, we reported the mean and standard deviation (SD). For categorical variables, we conducted chi-square tests to compare characteristics between those with vitamin D deficiency and vitamin D sufficiency; we conducted an independent samples *t*-test to compare the age between those with vitamin D deficiency and vitamin D sufficiency. We reported the prevalence of vitamin D deficiency (serum 25(OH)D concentration <50 nmol/L) in the total population and stratified by sex. We also reported mean serum 25(OH)D concentration by region of birth and season of blood collection.

Potential predictors were sex, age, region of birth, season of blood collection, BMI classification, smoking status, education category, physical activity category and socioeconomic status category. All variables were initially tested in bivariate Poisson regression models with robust variance based on the Huber sandwich estimate to obtain prevalence ratios (PR). Those variables with *p*-values < 0.20 were selected for inclusion in a single Poisson regression model. Variables were then eliminated in a stepwise procedure, and those changing the effect estimate of other potential predictors by <10% were excluded from the final model. All analyses were conducted using Stata version 14 [23].

## 3. Results

The majority (60%) of participants were born in sub-Saharan Africa, and the proportion of men and women was approximately equal (Table 1). More than 70% of participants were overweight or obese, and 65% had low/sedentary physical activity levels. Most participants (82%) were ex- or non-smokers. A total of 23% of participants were living in New South Wales, 20% in Western Australia, 17% in Queensland, 16% in Victoria, 9% in Australian Capital Territory, 8% in South Australia, <1% in Northern Territory and <1% in Tasmania.

**Figure 1:**
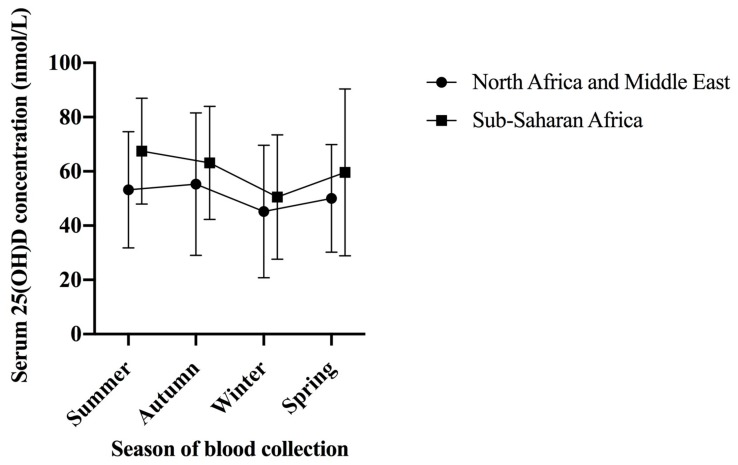
Mean (error bars ± 1 standard deviation) serum 25-hydroxyvitamin D concentration by season of blood collection in adults aged ≥18 years who were born in Africa and were included in the current study (North Africa and Middle East, *n* = 95; Sub-Saharan Africa, *n* = 141) in summer (December–February), autumn (March–May), winter (June–August) and spring (September–November).

**Figure 2:**
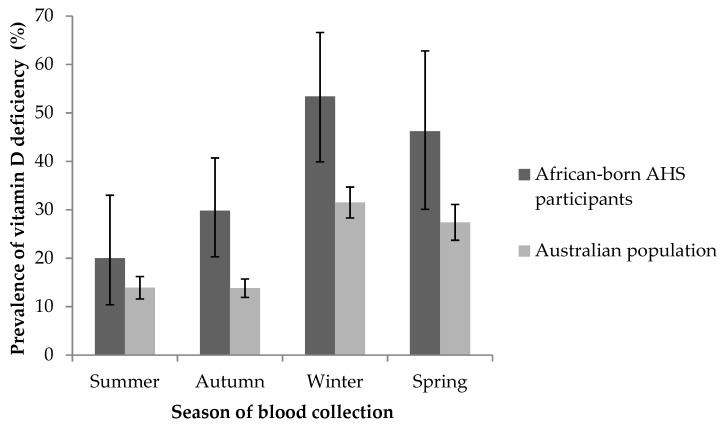
Prevalence (error bars 95% confidence interval) of vitamin D deficiency (serum 25-hydroxyvitamin D concentration <50 nmol/L) in African-born adults aged ≥18 years who participated in the Australian Health Survey (AHS) (*n* = 236) compared to the general Australian population.

The overall prevalence of vitamin D deficiency was 36% (Table 1), and the prevalence was similar for men and women (35% and 37%, respectively). Vitamin D deficiency was similarly prevalent in New South Wales (40%), Victoria (40%) and Western Australia (38%); we were unable to report prevalence of deficiency in other states/territories due to low numbers living outside of New South Wales, Victoria and Western Australia. Nearly one-third (29%) of participants born in sub-Saharan Africa were vitamin D deficient, compared with 46% of participants born in North Africa or the Middle East. Mean serum 25(OH)D concentrations were consistently lower in people born in North Africa or the Middle East than those born in sub-Saharan Africa, regardless of month of blood collection (Figure 1). The prevalence of vitamin D deficiency was consistently higher in African-born participants of the AHS compared to the general Australian population (Figure 2).

Bivariate models showed that age, region of birth, season of blood collection, BMI classification and physical activity category were associated with vitamin D deficiency at *p* < 0.20. After the elimination procedure, the final model included age, season of blood collection and physical activity category (Table 2). The prevalence ratio for vitamin D deficiency decreased by 2% per older year of age, and was 1.6 times higher in those with low/sedentary, compared to moderate/high, physical activity levels. The greatest risk was for those assessed during winter/spring compared with summer/autumn. The mean (SD) serum 25(OH)D concentrations were 51 (24) nmol/L (50% deficient) and 61 (23) nmol/L (26% deficient) for those assessed during winter/spring compared with summer/autumn, respectively. The mean (SD) serum 25(OH)D concentrations were 54 (23) nmol/L (41% deficient) and 62 (25) nmol/L (27% deficient) for those with low/sedentary, compared with moderate/high, physical activity levels, respectively.

## 4. Discussion

This study provides the first estimate of the prevalence of vitamin D deficiency in African immigrants living in Australia, which, at 36%, is substantially higher than that of the general Australian adult population at 23% [3]. A further 44% were vitamin D insufficient (25(OH)D 50 to <75 nmol/L). The AHS was not designed to generate a random sample of African-born people living in Australia. Characteristics of the small AHS sample of African-born people may thus differ from the total population of African-born adults living in Australia, and our results may not be generalisable to the total African-born population. Nevertheless, by comparing our AHS-based prevalence data with the census population data [24], we estimated that, of ~580,000 African-born Australian adult residents in 2011, approximately 209,000 and 464,000 could have serum 25(OH)D concentrations <50 and <75 nmol/L, respectively.

Our study is the largest, and the first, to examine the Australia-wide prevalence of vitamin D deficiency in African-born residents. Previous studies have been limited to single cities. Based on African immigrants living in Sydney and Melbourne, the prevalence of vitamin D deficiency has been reported as greater than 90% [4,5,6,7,8]. In Sydney, almost all (99%) study participants were deficient in winter [8]. Also, in Melbourne, 44% of African children had severe vitamin D deficiency (25(OH)D < 25 nmol/L), while 87% were vitamin D deficient (25(OH)D < 50 nmol/L) [6]. Compared with our study, the higher prevalence estimates in these previous studies may be due to location; Sydney and Melbourne have lower non-summer UV irradiance, lower total daily hours of sunshine [25] and lower temperatures, leading to less skin being exposed to the sun, compared with some other Australian cities (e.g., Perth, Brisbane). Furthermore, the use of non-certified assays may result in overestimation of the prevalence of vitamin D deficiency [26,27]. However, awareness of the greater risk of vitamin D deficiency amongst dark-skinned people appears to be improving [28].

In a study of adult immigrants from East Africa living in Melbourne in 2000, being female, Muslim and mostly covered were associated with increased risk of vitamin D deficiency in unadjusted models (odds ratio (OR) 10.1, 95% CI (4.2, 24.5) for female vs. male participants; OR 14.3, 95% CI (3.1, 131.9) for Muslim vs. Christian participants; and OR 11.1, 95% CI (4.6, 26.6) for being mostly covered vs. not) [4]. Similarly, in a survey of East-African children living in Melbourne in 2000–2002, wearing a head cover was associated with increased risk of vitamin D deficiency in an unadjusted model (risk ratio 2.4; 95% CI (1.9, 3.1)) [6]. However, there was no sex difference in the prevalence of vitamin D deficiency in our study, perhaps due to a shift in cultural clothing or lifestyle factors over time [29] or due to increasing use of vitamin D supplements by women. Compared with men, women are twice as likely to use vitamin D-containing supplements, and those born in Africa or the Middle East are more likely to use vitamin D-containing supplements than those born in Australia or New Zealand [30].

In our study, predictors of vitamin D deficiency shared with the general Australian adult population [3] included age, season of blood collection and physical activity. Lower prevalence of vitamin D deficiency in older adults may be a result of increased vitamin D supplement use in older age groups [30], while those who are less physically active may spend less time outside, decreasing their potential for sun exposure and vitamin D synthesis.

A qualitative study of African women living in Melbourne showed that lifestyle changes when moving to Australia, along with cultural roles, were predictors of vitamin D deficiency [28]. The women identified that living in high-rise buildings with no backyards led to lower sun exposure, and, unlike in Africa, they were not required to walk outdoors to buy fresh fruit and vegetables. Most women prioritised their culturally important role as caregiver over concerns for their own health and wellbeing. Hence, correcting vitamin D deficiency through diet or supplementation was not a priority, even when vitamin D supplements were readily available in the home. Increased duration of residence in Australia has also been identified as a predictor of vitamin D deficiency in African and Middle Eastern immigrants living in Australia [4,6,8].

A major strength of our study is the use of an assay certified to the RMPs for measuring serum 25(OH)D concentrations; this is the first time a certified assay has been used to assess vitamin D status in African people living in Australia. Some uncertified assays have been shown to return erroneously low 25(OH)D concentrations and, thus, a higher apparent prevalence of vitamin D deficiency [26,27]. A further strength was the comprehensive data on demographic and lifestyle characteristics included in the AHS, which allowed for potential predictors of vitamin D deficiency to be determined. However, we were not able to test associations between religion, sun exposure, longer residence in Australia, skin colour or cultural clothing habits (which have been noted as risk factors in other studies [4,6,7,8]) and risk of vitamin D deficiency.

## 5. Conclusions

We found a high prevalence of vitamin D deficiency (36%) among adult African immigrants living in Australia; hence, there is a need for culturally appropriate guidelines and messaging on safe sun exposure, dietary vitamin D and supplementation. However, promoting awareness and knowledge about vitamin D deficiency, such as providing resources through health professional services, may not be enough to result in behaviour change in this population group [28]. Rather, translation strategies to promote vitamin D sufficiency among African people living in Australia should be developed in collaboration with community groups to ensure they are culturally appropriate and meet the needs of the African immigrant population.

## Figures and Tables

**Table 1:** Characteristics of adults aged ≥18 years who were born in Africa and were included in the current study, in the total population (*n* = 236) and in those with vitamin D deficiency (*n* = 85) and vitamin D sufficiency (*n* = 151)

Characteristic	Total Population	Vitamin D Deficient	Vitamin D Sufficient	*p*-Value
	*n* (%)	*n* (%)	*n* (%)	
Sex				0.849
Men	113 (48)	40 (47)	73 (48)	
Women	123 (52)	45 (53)	78 (52)	
Age, years, mean (SD)	45.5 (15)	41.3 (15)	47.9 (15)	0.001
Region of birth				0.007
North Africa and Middle East	95 (40)	44 (52)	51 (34)	
Sub-Saharan Africa	141 (60)	41 (48)	100 (66)	
Season				<0.001
Summer/autumn (December–May)	139 (59)	36 (42)	103 (68)	
Winter/spring (June–November)	97 (41)	49 (58)	48 (32)	
Body mass index category				0.036
Underweight/healthy weight (<25 kg/m^2^)	68 (29)	26 (31)	42 (28)	
Overweight (25.0 to <30 kg/m^2^)	99 (42)	27 (32)	72 (48)	
Obese (≥30 kg/m^2^)	69 (29)	32 (38)	37 (25)	
Smoking				0.857
Ex/non-smoker	193 (82)	69 (81)	124 (82)	
Current	43 (18)	16 (19)	27 (18)	
Education				0.583
Less than university	111 (47)	42 (49)	69 (46)	
University	125 (53)	43 (51)	82 (54)	
Physical activity				0.032
Low/sedentary	154 (65)	63 (74)	91 (60)	
Moderate/high	82 (35)	22 (26)	60 (40)	
Socioeconomic status				0.101
Low	100 (42)	42 (49)	58 (38)	
High	136 (58)	43 (51)	93 (62)	
Vitamin D status ^1^				
Deficient (25(OH)D <50 nmol/L)	85 (36)	N/A	N/A	N/A
Insufficient (25(OH)D 50 to <75 nmol/L)	103 (44)	N/A	N/A	N/A
Sufficient (25(OH)D ≥75 nmol/L)	48 (20)	N/A	N/A	N/A

25(OH)D, 25-hydroxyvitamin D; N/A, not applicable. ^1^ Based on year-round blood collection, with each person sampled on one occasion only.

**Table 2:** Poisson regression model showing predictors of vitamin D deficiency (serum 25-hydroxyvitamin D concentration <50 nmol/L) in adults aged ≥18 years who were born in Africa and were included in the current study (*n* = 236).

Characteristic	PR (95% CI)	*p*-Value
Age, per year	0.98 (0.97, 0.99)	0.004
Season		
Summer/autumn	Ref	
Winter/spring	1.89 (1.35, 2.64)	<0.001
Physical activity		
Moderate/high	Ref	
Low/sedentary	1.64 (1.12, 2.39)	0.011

PR, prevalence ratio; CI, confidence interval; summer/autumn, December–May; winter/spring, June–November.
